# Visualization of endogenous enhancer-promoter interactions in a single nucleus through chromatin labeling

**DOI:** 10.1016/j.mocell.2024.100121

**Published:** 2024-10-08

**Authors:** Gunhee Park, Won-Ki Cho

**Affiliations:** 1 Department of Biological Sciences, Korea Advanced Institute of Science and Technology (KAIST), 291 Deahak-ro, Yuseong-gu, Daejeon 34141 Korea; 2 KAIST Stem Cell Research Center, Korea Advanced Institute of Science and Technology (KAIST), 291 Deahak-ro, Yuseong-gu, Daejeon 34141, Korea

**Keywords:** Chromatin labeling, Clustered Regularly Interspaced Short Palindromic Repeats, Enhancer-promoter interaction, MS2 tagging, Tetracycline operator-tetracycline regulator

## Abstract

Recent studies highlight the critical role of nuclear genome organization in regulating gene expression. Dynamic changes in the hierarchical structure of chromatin modulate transcription by influencing the recruitment of transcription factors and altering the epigenetic landscape. Among these regulatory mechanisms, enhancer-promoter (E-P) interactions are of particular importance. Enhancers physically interact with the promoters of target genes, a process mediated by various coactivators, which facilitates the transfer of enhancer-bound transcription factors and ultimately leads to transcriptional bursting. While next-generation sequencing techniques have provided significant insights into the features of E-P interactions, the effects of cell-to-cell heterogeneity and the physical dynamics of these interactions remain poorly understood due to the lack of spatiotemporal information. In this article, we introduce a platform that enables imaging-based approaches to visualize E-P interactions at the single-cell level.

## INTRODUCTION

The genome is organized into a hierarchical chromatin structure in the cell nucleus. Chromatin structures, including DNA loops, topologically associated domains, and compartments A/B, are known to regulate gene expression by modulating the accessibility of transcription factors through the insulation of in-cis elements or chromatin compaction (Misteli, 2020). A precise chromatin structure organization is required for supporting the development and maintaining cellular homeostasis. As many of gene mis-regulation–related diseases by topologically associated domain boundary alteration or chromatin translocation were reported, the importance of gene regulation mechanism within chromatin structure organization has emerged (Anania, 2020). With the advancement of next-generation sequencing technologies, such as Hi-Chromatin conformation capture and Chromatin Interaction Analysis by Paired-End Tag Sequencing, which elucidate chromatin contacts, a deeper understanding of the 3D chromatin structure has begun to emerge (Kempfer and Pombo, 2020, McCord et al., 2020). However, sequencing-based approaches, which rely on the fixation of millions of cells during library preparation, yield ensemble average data and lack cell-to-cell heterogeneity and spatiotemporal information (Hwang, 2023). This limitation underscores the need for research at the single-cell level to investigate how chromatin dynamics operate in a spatiotemporal manner.

Enhancer-promoter interaction is a well-studied mechanism by which chromatin structure regulates gene expression (Schoenfelder and Fraser, 2019). Enhancers physically contact promoters through DNA loop extrusion, a process mediated by various proteins such as coactivators and mediators (Cho et al., 2018, Shin, 2022). During this process, transcription factors recruited to enhancers are transferred to promoters, facilitating transcription.

Although the molecular mechanisms of enhancer-promoter interactions have been extensively studied through sequencing-based approaches, the spatiotemporal dynamics of these interactions remain elusive. To investigate the real-time contact frequency and duration of enhancer-promoter interactions, as well as how these dynamics influence mRNA transcription bursting, spatiotemporal approaches at the single-cell level are required.

In the last decade, various technologies for fluorescently labeling chromatin have been developed (Shaban and Seeber, 2020). One technology is based on the Clustered Regularly Interspaced Short Palindromic Repeats system, utilizing dead Cas9 (dCas9) in conjunction with single-guide RNA (sgRNA) to bind target sites (Chen et al., 2013). This allows for fluorescent labeling of chromatin when dCas9 fused with a fluorescent protein is used. Significant optimization has been achieved to enhance efficiency and obtain a high signal-to-noise ratio (Ma et al., 2018). Moreover, advancements have been developed to overcome the limitations of Clustered Regularly Interspaced Short Palindromic Repeats–based technologies, which were previously restricted to labeling pre-existing repeat sequences. New methods involve knocking in repeat sequences at desired loci and using fluorescently tagged proteins that bind to specific sequences (Tasan et al., 2018).

Studies over the past few decades, on the other hand, have focused on fluorescent labeling of mRNA or specific proteins through aptamer-based single-molecule RNA labeling, which relies on RNA secondary structures that specific molecules or proteins can bind to (Hoppe and Ashe, 2021, Tutucci et al., 2018). The virus–derived aptamer MS2 binding sequence can be inserted into the 3′UTR of mRNA, allowing the MS2 coat protein (MCP) fused to a fluorescent protein to bind, thereby visualizing transcriptional bursting of nascent transcripts.

We introduce here a practical method to fluorescently label chromatin loci corresponding to enhancers and promoters combining previously established labeling systems, enabling real-time visualization of enhancer-promoter dynamics in a single-cell nucleus.

## DESIGN OF DCAS9 BINDING ARRAY

We designed the binding array to enhancer or gene-specific labeling with dCas9 system by gene synthesis. Binding array consists of 30 repeats of 20nt of target sequence followed by 3nt of Protospacer adjacent motif (PAM, NGG) sequence which can be bound by dCas9 with sgRNA (Carroll, 2023). “N” sequences for each PAM sequence were alternately A, T, G, and C. Target sequence was employed from endogenous sequence harbored in genomic region near the site that array will be knocked-in to avoid off-target labeling, and different target sequences were utilized on enhancer-specific array and gene-specific array, respectively. Each target sequence is apart 80nt of gap from each other to achieve sufficient dCas9 binding footprint.

In the upstream of target sequence repeat, GFP or BFP expression construct including CMV promoter and poly-A signal to positive cell selection after knock-in. Fluorescence protein (FP) expression construct and dCas9 binding array were flanked by 500 bp of homology arm.

## KNOCK-IN OF THE ARRAY INTO ENHANCER AND PROMOTER

We choose the MYC gene and its 515-kb upstream superenhancer that are well-defined enhancer-promoter interaction in colorectal cancer cells (Schuijers et al., 2018). We found the knock-in site around 1 kb upstream of the gene and downstream of the superenhancer, respectively, and designed the sgRNA for knock-in by IDT custom crRNA design tool (www.idtdna.com/site/order/designtool/index/CRISPR_CUSTOM) which showing highest on-target and off-target score. We cloned the designed sequence at the *Streptococcus pyogenes* Cas9 vectors (pSpCas9 (BB)-2A-Puro (PX459) V2.0) (Addgene #62988) as previously described (Ran et al., 2013).

**Fig. 1:**
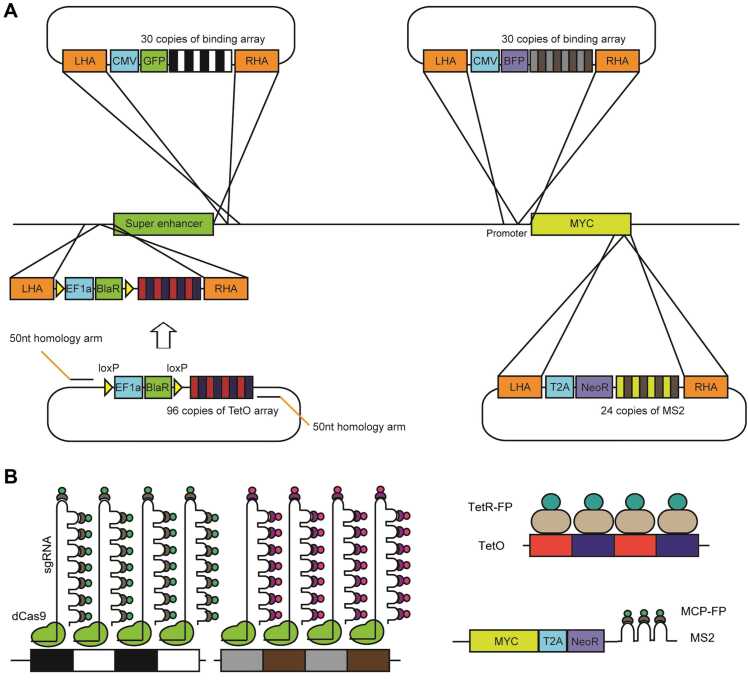
Schematic outline of the knock-in strategy for generating an enhancer-promoter-labeled cell line. (A) A dCas9 binding array, TetO array, and 24xMS2 are inserted into the superenhancer or the 3′ UTR of the MYC gene, respectively. (B) The fluorescence labeling principle of the dCas9 binding array, TetO array, and 24xMS2. dCas9 binds to the target site with sequence-specific, aptamer-tagged sgRNAs. The aptamer in the sgRNAs is orthogonally bound to FP-fused aptamer-binding proteins (left). The FP-fused TetR binds the TetO array, and the FP-fused MS2 coat protein binds 24 copies of the MS2 aptamer in the 3′ UTR of the mRNA (right).

We cotransfected the repair template of 1 μg of dCas9 binding array with FP expression construct and 1 μg of pX459 vector into 70% confluency of HCT116 cells on 6-well plate (Fig. 1A). The positive cells were sorted with fluorescence-activated cell sorting (FACS) at 48 hours after transfection. Positive cells were confirmed by fluorescence check with microscope, and genotyping with genomic DNA PCR.

## STABLE CELL LINE GENERATION EXPRESSING DCAS9

To generate stable dCas9–expressing cell line, we produced lentivirus–harboring dCas9 expression vector. Lentiviral expression vector for dCas9 with blastacidin selectable marker was transfected into HEK293 cells with corresponding packaging vector (pSPAX2) and envelop vector (pMD2.G). Viral media was harvested at 24 and 48 hours after transfection and filtrated followed by purification with PEG-it virus precipitation solution (System Biosciences) according to manufacturer’s protocol. Virus that precipitated in PBS was transduced on 70% confluency of HCT116 in 10 μg/mL in cell culture media. Twenty-four hours after transduction, cells were selected with 10 μg/mL blastacidin for 1 week. Positive cells were confirmed by genotyping with genomic DNA PCR.

## TRANSIENT EXPRESSION OF APTAMER-TAGGED SGRNA AND FP-FUSED APTAMER-BINDING PROTEIN

**Fig. 2:**
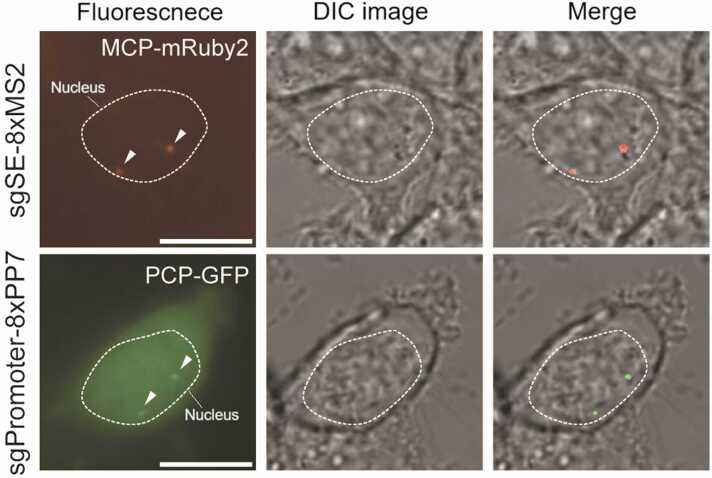
Live-cell imaging of cells harboring a dCas9 binding array, transfected with sgRNAs targeting each array and FP-fused aptamer-binding proteins. Scale bars represent 10 µm.

For multiplexed labeling of enhancer array and gene array at the same time, we utilized the sgRNA which is tagged with orthogonal aptamer sequence, such as MS2 and PP7 (Fig. 1B). sgRNA sequence targeting each array was cloned into MS2 or PP7-tagged sgRNA expression vector, respectively. Aptamer-tagged sgRNA expression vector was cotransfected with corresponding FP-fused binding protein expression vector, such as MCP-mRuby2 or PCP-mNeonGreen, into 50% to 70% confluency of cells on 35-mm glass bottom confocal dish (Cellvis). Transfected cells were imaged on fluorescence microscopy at 24 hours after transfection (Fig. 2).

## REPEAT SEQUENCE AND APTAMER-BASED LABELING

To visualize the nascent RNA expression on gene locus, we utilized the aptamer-tagging system on mRNA 3′UTR. Also, we alternatively employed the insertion of 96 copies of tetracycline operator (TetO) repeat array into the enhancer region of the MYC and expressed tetracycline regulator (TetR)-GFP specifically binding the TetO (Fig. 1B).

We amplified the TetO array repair template harboring blastacidin selectable marker by PCR with 50 bp of homology arm including primer and TetO array plasmid (Addgene#118713) as template. Amplified repair template was purified in distilled water. We cloned the sgRNA designed by previously described strategy into pX459 vector.

The repair template backbone plasmid harboring homology arms corresponding to 3′UTR of MYC gene was synthesized by gene synthesis. T2A-NeoR-24xMS2 construct was cloned between each homology arm. pX459 vector expressing the sgRNA targeting the site near stop codon of MYC was cloned with same strategy above.

TetO array for enhancer labeling was inserted first, followed by antibiotics selection with 10 μg/mL blastacidin for 1 week. About 70% confluency of HCT116 cells were transfected with 1 μg of purified linear repair template and 1 μg of pX459 vector. After selection, survived cells were sorted in 96-well plate as single cell by serial dilution to make monoclones. Successful insertion of array in each monoclones was confirmed with genomic DNA PCR.

TetO array harboring monoclone was transfected with 1 μg of MYC 3′UTR T2A-NeoR-24xMS2 repair template vector and 1 μg of pX459 vector. Transfected cells were selected with 1 mg/mL G-418 for 1 week, followed by sorted in 96-well plate as single cell to find in-cis double knock-in clone.

**Fig. 3:**
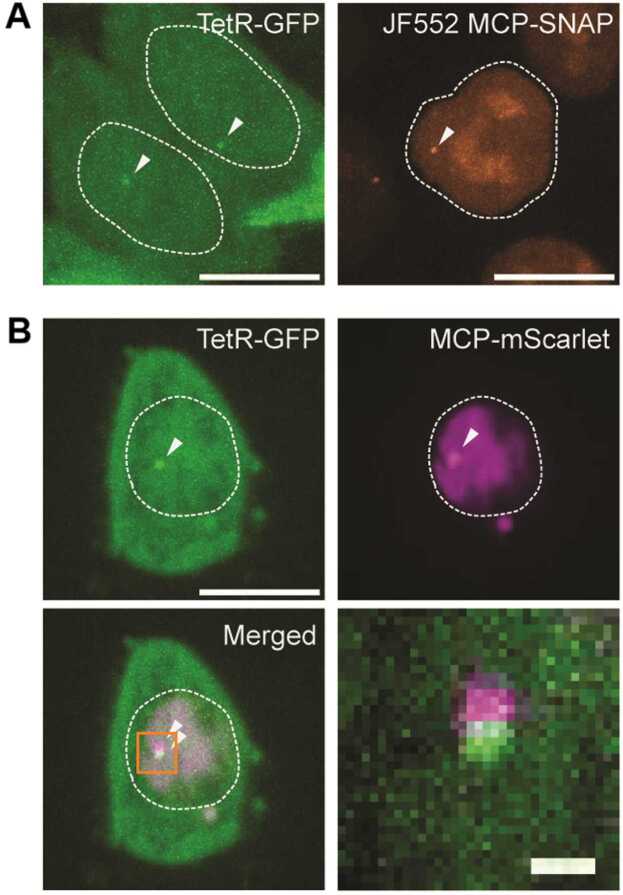
(A) Live-cell imaging of cells stably expressing TetR-GFP and MCP-FP, harboring 96 copies of TetO and 24 copies of MS2 in the superenhancer or 3′ UTR, respectively. (B) Dual-color live-cell imaging of the superenhancer and nascent transcription loci of *MYC*. Scale bars represent 10 µm, and 1 µm for zoom-in image.

To express the MCP-SNAP for MS2 labeling, 1 μg of MCP-SNAP cloned in PiggyBac vector was transfected with 1 μg of Super PiggyBac transposase expression vector (System Biosciences). Transfected cells were cultured more than 1 week to let the vectors be integrated into genome and transiently remained vectors be degraded. Cells were stained with 100 nM of JF552-SNAPtag ligand for 10 minutes and washed with fresh cell culture media, followed by FACS sorting of positive cells (Fig. 3A, right pannel). MCP-SNAP positive cells were transduced with lentivirus-containing TetR-GFP-expression vector (Fig. 3A, left pannel), packaged by same strategy described above, followed by FACS sorting of positive cells (Fig. 3B).

## Author Contributions

**Won-Ki Cho:** Writing—review and editing, Validation, Supervision, Resources, Project administration, Funding acquisition. **Gunhee Park:** Writing—original draft, Visualization, Validation, Methodology, Investigation, Formal analysis, Data curation, Conceptualization.

## Declaration of Competing Interests

The authors declare that they have no known competing financial interests or personal relationships that could have appeared to influence the work reported in this paper.
